# Non-Metallic Plastic Materials for Filling Teeth

**Published:** 1890-03

**Authors:** Otto Arnold

**Affiliations:** Columbus, Ohio.


AMERICAN JOURNAL

—OF—

DENTAL SCIENCE.

VOL. XXIII. BALTIMORE, MARCH, 1890. No. 11.

ARTICLE I.

### NON-METALLIC PLASTIC MATERIALS FOR FILLING TEETH.

BY OTTO ARNOLD, D. D. S., COLUMBUS, OHIO.

[Read before the Mississippi Valley Dental Association, at Cincinnati, Ohio, March, 1890.]

Of all operations in dentistry, that of filling teeth engages our consideration more extensively than all the rest. With rare exception, the largest part of an intelligent and well disposed dentist's practice is of this character.

There is a natural demand for this class of operations. People reluctantly consent to the loss of their teeth. As a rule, they prefer to retain their natural organs if conditions of health, comfort and usefulness can be secured.

This being the case, the question of filling materials becomes an important one for consideration.

*Gold and Amalgams.* The former, as yet, the peerless element for filling; the latter, so much abused and persecuted, but most excellent compound and second only to the former in honest and skillful hands—these are to-day the only substances in general use that may safely be called permanent filling materials, excepting platinum and platinum iridium foils that are used to a limited extent for special purposes only. I shall not further discuss either of these, but confine myself to the non-metallic plastics.

Scientific research and inventive genius of man has been taxed to the utmost for lo! these many years, in efforts to discover or prepare a plastic substance possessing properties essential to a perfect filling for teeth, viz.: adaptability; and above all requiring only a mediocre degree of manipulative ability for its use. It is useless to state that this ideal filling is yet to be discovered.

To the unsuspecting and susceptible in our profession, many tempting morsels are offered by the numberless preparations in the market claiming to meet all these requirements. Likewise are the public falsely impressed by the euphonious, but misleading adjectives under which most of them all; for example, osteo-plastic, diamond cement, os-artificial, plastic bone, porcelain cement, etc., etc., *ad infinitum.* Many, if not all of these preparations, have most desirable properties; some excelling in one particular, some in another. In the aggregate they are useful and I may say indispensable; but as permanent filling materials they are, individually and collectively, failures.

The employment by the earlier dentists of gums, as mastic and sandarac, in etherial and alcoholic solutions for the stopping of cavities of decay, is the first approach history records of plastic fillings. About the year 1848, however, the first substantial progress was made in this direction by the use of gutta-percha as a temporary filling material; this was soon recognized as an important element, and before long came into universal use and favor. A little later the well-known compound, Hill's Stopping, was introduced, which is a modification of gutta-percha by the addition of certain mineral elements to make it harder, therefore more available for permanent fillings. This substance filled a long-felt want, held its own for many years, and is used to-day by a large class of good operators.

About thirty years ago oxychloride of zinc was introduced, the first of a now well-known class of filling materials, viz., the zinc plastics. Next in order came oxyphosphate of zinc, followed by innumerable modifications and combinations, all included in this class now so extensive that a chronological order of description would weary the most patient auditor.

It is not the purpose of the writer to detail the chemical composition of these products, or recommend any special brand, but I propose to speak of their characteristics as a class, their practicability and general value in dental practice at the present time.

It can not be denied that the introduction of gutta-percha and the zinc plastics was the beginning of a new era in operative dentistry that made it possible to attain results never before brought about. Prior to that time little, if anything, had been accomplished in the direction of protecting pulps from the effects of thermal irritation. The solution of this problem alone is of such intrinsic worth as to make any material capable of contributing to that end of inestimable value. All preparations of the zinc plastics, likewise gutta-percha, at least so far as the writer has knowledge, are more or less non-conductors of caloric, therefore valuable for this purpose; and from the extensive knowledge on this subject, it is almost an unpardonable offense to ignore their use in all large cavities as a protection to the pulps. These conclusions are from a practical point of view and with due deference to the experiments with the electric thermostat which indicates the contrary to be the case.

Gutta-percha, however, unless in solution of chloroform or other volatile solvents, is not wholly safe, unless the greatest care is exercised to prevent its introduction into the cavity in too heated a condition. This is a serious obstacle, as the minimum degree of heat necessary to plasticity may, especially if the pulp is near the surface, be sufficient to permanently injure this organ. The pressure generally necessary to adapt this substance to place is another objection. So nothing short of the greatest caution in its use will give certain results. Gutta-percha as a filling material, compared with the zinc plastics for inside use, and amalgam for outer surfaces, has a limited sphere of usefulness. This deduction takes into account our present available facilities for tooth repair and restoration by crowning, and is from the standpoint, if you please, of 1890.

All filling materials are in their nature foreign to tooth material. Therefore, the substance that nearest approximates the latter in physical characteristics, comes nearest being the perfect filling. In this respect the zinc plastics are as yet in the lead. Their ease of manipulation, perfect adaptation without pressure, non-contractability, freedom from heat, wholesome therapeutic action and hardness, are qualities peculiar to themselves; it seems nothing further is needed to make the ideal filling except indestructibility.

Each of these preparations belong to either one or the other of two classes, viz., oxychlorides, and oxyphosphates of zinc and their possible modifications. The oxychlorides have an escharotic action on organized tissue which makes them unsafe as nerve cappings *per se*; but when used in connection with an intervening layer of a non-irritant, they become useful aids for this purpose. They are decidedly antiseptic but readily soluble in oral fluids, and are distinguished as "the most preservative and at the same time the most perishable" of all filling materials. The antiseptic quality is a valuable feature for root fillings, and as these are supposed to be protected from the fluids of the mouth, their solidity in this relation is unimportant.

The zinc phosphates are less irritating in their action on organized tissue, are denser in structure and less soluble in the oral fluids, and for general purposes are preferable and in more general use than the zinc chlorides.

Briefly, then, to sum the matter up, what is the value of zinc plastics in dental practice, and to what extent should they be used? The operator being more or less familiar with the working qualities and merits of these preparations ought to be able ro intelligently select the most suitable for cases in hand.

All large cavities should have a layer of this substance intervening between metallic fillings and their deeper portions, if possible, to protect the pulp from thermal irritation. This seems like an uncalled for statement, but the importance of the principle is sufficient to bear emphasizing, for there are some operators, even in this day and generation, who are indifferent in this direction, unless actual exposure of the pulp has occurred. The absence of visible signs of pulp exposure is not always to be relied upon as a guide; then cavities may be so obscurely located that a satisfactory view of their interior is impossible. In view of these elements of uncertainty—where the hazard is great—chances should be reduced to the minimum. As a covering contiguous to exposed pulps, the more neutral and non-irritating of these preparations possess more good qualities than any other substance, chiefly on account of their adaptation without pressure and the non-generation of heat.

For filling root canals, zinc plastics are unsurpassed. The method I have practiced for a long time, with more satisfactory results than any other, is to carry these to the apex on shreds of cotton of a fineness suitable to the case in hand, using necessarily the non-sticky variety. The facility and greater certainty with which the apex may be reached, combined with the imperviousness and antiseptic properties, make them the ideal root filling. For use in connection with crown and bridge-work, we have nothing to compare with them and can only say that they stand alone. For entire fillings in teeth that promise pathological complications, or for obvious reasons require temporary operations, they are a most valuable material. Taking them all in all, they occupy an important place in dentistry, and we could ill afford to return to the methods in vogue before their introduction.

But like all good things zinc plastics are often abused and their use is not always followed by the best results. The grateful sense of comfort following their introduction into sensitive cavities affords too great an inducement for their use in cases demanding something more permanent. This property is too often taken advantage of for hastily terminating a disagreeable engagement rather than subserving the best interests of the patient. For the time being the patient is satisfied; but when, in a few months perhaps, the filling has appreciably disintegrated, there is disappointment and a diminished regard for dental principles in general. The operator who has so little control over his patient that he can not do thorough work at once in simple but sensitive cavities, will probably accomplish little more at future sittings. I am opposed to temporary fillings as a substitute for something better, except possibly in children's cases, or where pathological or certain sexual conditions prohibit.

But the principal provocation for criticism is the indiscriminate practice of prostituting a good thing for uses other than its proper one. Zinc plastics are used to a large extent for front teeth, and recommended for permanent work, under such significant but deceptive names as bone fillings, porcelain fillings, etc., etc. The outcome of such practice can result only against the general good of the profession through the ultimate disappointment and loss to the innocent victim.

The remedy that suggests itself against such abuse is to be more explicit in imparting advice on these matters. We are consulted as authorities on these subjects by a confiding public; let us see to it that we enlighten them as to the facts in the case and fearlessly uphold the right. When temporary fillings must be inserted impress the patient forcibly as to their limited utility. If such fillings are preferred on account of inexpensiveness or for any other reason, be emphatic in calling them temporary fillings, and nothing more. It is our duty to denounce in no uncertain terms all doubtful practices that tend to reflect upon our profession. An exposition of disreputable methods in vogue for selfish gains will do no honest person harm; but, on the contrary, so strengthen public confidence as to work lasting benefit to both patients and conscientious operators, while all doubtful methods will be left to their true sphere —the office of the enterprising advertiser of nostrums and miraculous devices.—*Dental Register.*

